# The Effect of Washing, Blanching and Frozen Storage on Pesticide Residue in Spinach

**DOI:** 10.3390/foods12142806

**Published:** 2023-07-24

**Authors:** Federica Flamminii, Silvia Minetti, Adriano Mollica, Angelo Cichelli, Lorenzo Cerretani

**Affiliations:** 1 Department of Innovative Technologies in Medicine and Dentistry, University “G. d’Annunzio” of Chieti-Pescara, Via dei Vestini, 66100 Chieti, Italy; angelo.cichelli@unich.it; 2 Società Abruzzese Lavorazione Prodotti Agricoli (S.A.L.P.A.) S.A.C.arl, Via Nazionale, 64026 Roseto degli Abruzzi, Italy; silvia.minetti@salparoseto.it (S.M.); lorenzo.cerretani@salparoseto.it (L.C.); 3 Department of Pharmacy, University “G. d’Annunzio” of Chieti-Pescara, Via dei Vestini, 66100 Chieti, Italy; a.mollica@unich.it

**Keywords:** spinach, household processing, washing, blanching, pesticide residues, processing factor, frozen storage, propamocarb, lambda-cyhalothrin, propamocarb n-desmethyl

## Abstract

Spinach (*Spinacia oleracea* L.) is a representative green leafy vegetable commonly consumed fresh or as a ready-to-cook frozen product, with increasing consumption because of its many health-related properties. Among leafy vegetables, spinach poses a major concern in terms of pesticide residue detection due to common phytotechnical practices. In this study, spinach leaves were treated in the open field with three commercial pesticide formulations containing propamocarb, lambda-cyhalothrin, fluopicolide and chlorantraniliprole at the highest concentration. The effects of the successive processing steps of washing, blanching, freezing and frozen storage were evaluated on the levels of the four pesticide residues and the degradation product (propamocarb n-desmethyl). The washing step caused a reduction of fluopicolide and chlorantraniliprole of −47% and −43%, respectively, while having a mild effect on lambda-cyhalothrin content (+5%). A two-minute blanching step allowed for the reduction of pesticides content ranging from −41% to −4% with respect to the washed sample. Different behaviors were depicted for longer blanching times, mainly for propamocarb, reaching −56% after 10 min of treatment. Processing factors higher than 1 were reported mainly for lambda-cyhalothrin and fluopicolide. Frozen storage led to a slight increase in the pesticide content in samples treated for 6 and 10 min. The optimal blanching treatment for spinach, submitted to freezing and frozen storage, seems to be 2 min at 80 °C.

## 1. Introduction

Global demand for food is ever increasing and accompanied by changes in lifestyles and choices of food. To feed the ever-growing global population, it is estimated that global food production will need to increase 70% by the year 2050, and the wide use of pesticides in the agricultural sector in some way is encouraged to boost crop production [1].

Pesticides, referred to also as plant protection products (PPPs), are globally used in agriculture systems to control plant diseases, insect pests and weeds, providing high yields and product quality [2]. Despite the positive effects on agricultural production, there are many ecological and health risks associated with pesticides and their residues in foodstuffs. Indeed, toxic effects on humans, ranging from short-term effects such as headaches and nausea to chronic effects like cancer, reproductive damage, endocrine disruption or autism, learning disabilities and neurological disorders, including Parkinson’s and Alzheimer’s diseases, have been frequently reported [3,4,5]. The issue of food contamination caused by pesticide residues is becoming increasingly important due to the growing global demand for food. The European Union introduced several policies regulating the use of PPPs, namely, Regulation (EC) n. 1107/2009 and Regulation (EC) n. 396/2005, concerning the placing of plant protection products on the market and the maximum residue levels (MRLs) of pesticides in/on food and feed of plant and animal origin, respectively [6,7]. Along with the promotion of organic farming, the adoption of integrated pest management (IPM) represents one of the tools for low-pesticide-input pest management and one of the major pathways for progress in reducing pesticide risks. It offers a framework for managing economic, health and environmental risks while minimizing undue outcomes for crop production [8].

The persistence of pesticides in plants varies depending on environmental conditions that lead to their hydrolysis, oxidation, reduction, photolysis [9] and the alteration of their properties such as solubility, volatilization, dissociation constant and formulation; moreover, their persistence induces changes in vegetable characteristics such as plant morphology and metabolic activity. Propamocarb (propyl [3-(dimethylamino)propyl]carbamate), for example, is a systemic fungicide with protective action against phycomycetous diseases. It is known to degrade into a variety of metabolite as propamocarb hydrochloride, n-oxide propamocarb, oxazoline-2-one propamocarb, 2-hydroxypropamocarb and n-desmethyl propamocarb [10]. Despite the fact that metabolites can be more toxic than the parent compound, little is known about the dissipation of both the parent and metabolite compounds, and the toxicity of metabolites has not been studied yet [11]. Lambda-cyhalothrin, a nonsystemic and lipophilic active substance which belongs to the group of pyrethroid compounds, is a broad-spectrum insecticide that acts on the nervous system, resulting in a product that is highly stable to light and high temperature and has a half-life of seven days [12].

Agricultural products are generally consumed after being processed. Washing represents the first and most common domestic or industrial unit of operation, useful to remove surface pesticide residues in fruits and vegetables [13], and major portions of polar compounds [14]. Wu and co-authors investigated the effect on different agricultural products of different home/commercial washing strategies for pesticide removal, showing that sodium bicarbonate solution, ozone water and active oxygen solution were more effective in decontamination of spinach than in kumquat and cucumber [13]. Washing with diluted salt or/and chemical solution can be a more effective decontamination strategy than the use of pure water [15].

Further processing steps such as peeling, blanching, baking, pasteurization, frying and different operation-unit combinations are identified as critical processing operations and significantly reduce pesticide residues by up to 100% removal. Peeling or trimming the outer skin of some fruits and fruiting vegetables represents the most efficient approach to reducing pesticide residues [13]. Hot-water blanching increases pesticide removal and may hydrolyze substantial fractions of nonpersistent compounds [14]. Numerous factors impact the level and degradation of residues after the cooking process; these include time, temperature, pH, a decrease in moisture content and the type of cooking system (open or closed). Blanching, as a single operation, was recognized to be the most effective treatment to reduce boscalid (45%) and propamocarb (72%), enhancing the elimination of the residues in spinach leaves [4].

Although broadly studied and well-established as decontamination methods, the aforementioned procedures are deleterious to the physical and phytochemical (flavonoid, carotenoid, etc.) and micronutrient content of fruits and vegetables [9]. The recent approaches in sanitation of fruits and vegetables, based on the oxidation processes (generation of free radicals such as hydroxyl radicals), include modern nonthermal techniques such as ozonation, ultrasonication, high hydrostatic pressure (HHP), electrolyzed water (EW), gamma radiation, and nonthermal plasma technology, which avoid the undesirable effects of heat treatments [16,17].

Despite the positive effects of technological treatments, some toxic compounds or metabolites may be formed during processing, and in some cases, processes can induce an increase in pesticide residues [2]. Different studies have been performed to measure the concentrations of pesticide residues after home or industrial processing. Processing factors (PFs) indices for pesticides in foods, related to the ratio between residue concentrations in the processed commodity and the same in the raw material, represent the effect of cumulative processes on the residues levels [18]. These PFs indices highlight the higher reduction in pesticide concentrations as a result of washing, blanching and sterilization than in washing, blanching and microwave cooking [4]

Human consumption of green leafy vegetables, which are cheap and easily accessible, has increased worldwide because they offer multiple health benefits due to the presence of vitamins, minerals, fiber, essential amino acids, polyphenols and flavonoids [2].

Among leaf vegetables, spinach (*Spinacia oleracea* L.) is commonly consumed fresh in salads, soups or as a ready-to-cook frozen product. This leafy vegetable poses a major concern in terms of pesticide residue detection, in relation to common phytotechnical practices [4,19], and its morphology—indeed, the morphology of all leafy vegetables including spinach–means it is not peeled and is difficult to clean and, only attached dust, insects and outer substances can be removed [20]. In addition, because of the large surface area of the leaves, pesticide residues are likely to remain on them [20] and in some cases, such as in the drying process, the residue content can increase as a result of the water loss during processing [13] or lead to the production of more toxic products or metabolites [21].

The abundance of agricultural commodities at a reasonably low cost is related to the use of pesticides, which are able to increase the production yield; this abundance, along with, consumer demand for a wide variety of products and the desire for them to be available the whole year long, has stimulated the development of frozen products that can generally be found worldwide in retail [22]. Despite numerous studies concerning the effects of home/commercial processes on the pesticide residue in foods, few research studies have focused on the effect of frozen storage on the pesticide residue content in foods, in particular zucchini, tomato, apple, mango and fish, where controversial results have been observed [23,24,25,26,27,28].

Therefore, based on this scenario, the aim of this research is to study the effect of the washing and blanching processes on the residual content of chlorantraniliprole, lambda-cyhalothrin, fluopicolide, propamocarb and propamocarb n-desmethyl in spinach. Furthermore, the effect of freezing and frozen storage (−20 °C) is also evaluated during a period of ten months.

## 2. Materials and Methods

### 2.1. Materials

HPLC-grade methanol (MeOH) and acetonitrile (ACN) were purchased from Honeywell (Charlotte, NC, USA); and formic acid (purity 99.7%) and ammonium formate (purity 99%) were purchased from Sigma-Aldrich (St. Louis, MO, USA). The QuEChERS extraction kit, which contains 4 g of magnesium sulfate (MgSO_4_), 1 g of sodium chloride (NaCl) and dispersive SPE (dSPE), and the cleanup kit tubes were obtained from Interchim (Montlucon, France). Analytical reference standards of fluopicolide, propamocarb hydrochloride, propamocarb n-desmethyl, chlorantraniliprole and lambda-cyhalothrin compounds were purchased from Labinstruments (Castellana Grotte, Bari, Italy).

### 2.2. Application of Pesticides and Harvesting

Spinach (*Spinacia oleracea*) var. Monterey was cultivated in a commercial open field located in Corropoli (Teramo, Italy) (42°49′29.4″ north latitude, 13°53′07.9″ east longitude). Different commercial pesticide formulations—such as VOLARE^®^ (Bayer AG, Leverkusen, Germany), composed of 5.53% (*w*/*w*) fluopicolide and 55.3% (*w*/*w*) propamocarb hydrochloride; ALTACOR^®^ (FMC Agro Italia Srl, Bergamo, Italy) composed of 35% (*w*/*w*) chlorantraniliprole; and KARATE ZEON^®^ (Syngenta Crop Protection AG, Basil, Switzerland) with 9.48% (*w*/*w*) lambda-cyhalothrin—were nebulized at the highest concentration defined by the manufacturers on 4/5 spinach leaves. Physicochemical characteristics of the main active compounds contained in each commercial preparation are reported in Table 1. Fresh samples of spinach leaves were manually harvested two days (48 h) after the treatment, in order to have the highest concentration of pesticides, and were carried to Società Abruzzese Lavorazione Prodotti Agricoli (S.A.L.P.A.) S.A.C.arl industry (Roseto degli Abruzzi, Teramo, Italy), which was involved in this research, for the processing treatments, storage and analysis steps.

### 2.3. Methods of Washing, Blanching and Freezing

**Figure 1:**
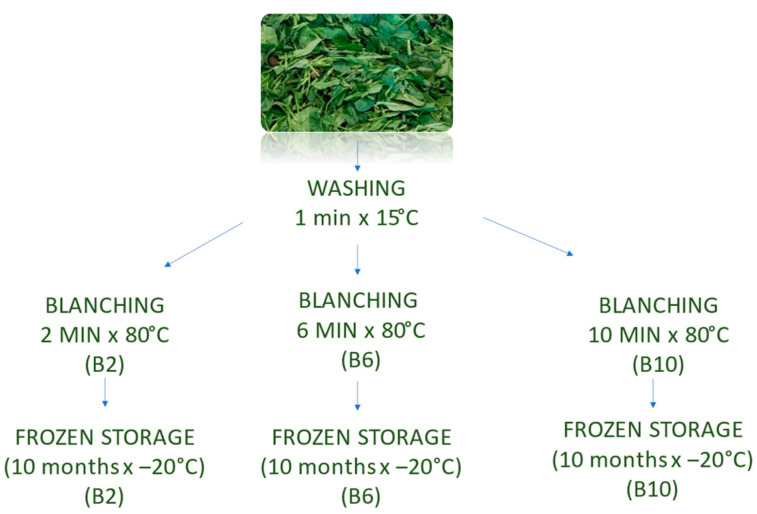
Flow diagram of spinach processing steps and samplings.

Fresh spinach samples (F) were harvested, divided into different batches (a, b, c) and subjected to successive processing steps, as shown in the process flowchart (Figure 1). The washing step (W) was conducted with running tap water (15 °C for 1 min); subsequently, each sample batch was independently blanched with hot water (80 ± 1 °C) in a 1:2 ratio. Different heat treatment timings were defined: 2 min (B2), 6 min (B6) and 10 min (B10). At the end of the treatments, each sample was lightly drained, placed into square shapes, frozen with dry ice (CO_2_) and stored at −20 °C for further analysis during storage time at T1 (day after the treatment), T2 (after 4 months) and T3 (after 10 months).

### 2.4. Extraction and Purification of Pesticides

The extraction of pesticides was performed on fresh and frozen samples. In the first step, spinach samples were comminuted using the lab knife mill Retsch GM 200 (Haan, Germany). All equipment was carefully cleaned and rinsed with water between the processing of each sample. The extraction was performed using the QuEChERS (quick, easy, cheap, effective, rugged and safe) method. An amount of homogenized sample (10 ± 0.1 g) was weighed into a 50 mL extraction centrifuge tube and filled with 1 g of sodium chloride and 4 g of anhydrous magnesium sulfate; then, an addition was made of 10 mL of a solution of 0.02% (*v*/*v*) formic acid in acetonitrile with bromo dimethylphenyl carbamate (BDC) and triphenyl phosphate (TPP), as internal standards. The mixture was shaken for 20 min and then centrifuged for 5 min at 5000 rpm. An aliquot of 3 mL of supernatant was transferred into a 15 mL centrifuge tube for the cleanup step, which contained 25 mg primary secondary amine (PSA), 2.5 mg graphitized carbon black (GCB) and 150 mg magnesium sulfate (MgSO_4_). PSA sorbent removes polar inferences including organic acids and sugars; GCB removes pigments such as chlorophyll and carotenoids; and magnesium sulfate adsorbs water. The tubes were shaken for 5 min and centrifuged for 5 min at 5000 rpm. An amount of 200 microliters of supernatant was diluted with 800 mL of ultrapure water or acetone directly in the vial for LC or GC analysis, respectively.

### 2.5. LC-MS/MS Analysis

Determinations of pesticide residues were performed in a Waters XEVO TQ-S micro HPLC (Waters, Milford, MA, USA) equipped with a reversed-phase column (KINETEX C18, 2.6 μm, 100 mm × 2.1 mm, Phenomenex, Torrance, CA, USA) at 40 °C. The mobile phase used was water/methanol (80:20) (containing 0.1% formic acid and ammoniac 0.02%) as phase A and methanol (containing 0.1% formic acid and ammoniac 0.02%) as phase B, with a flow rate of 0.4 mL min^−1^. The injection volume was 10 μL. For the mass spectrometric analysis, an XEVO triple quadrupole LC/MS system was applied. The ESI source was operated in positive ionization mode, and its parameters were as follows: source temperature, 150 °C; gas desolvation temperature, 600 °C; cone gas flow, 17 L h^−1^; desolvation gas flow, 1000 L h^−1^; nitrogen gas used as the nebulizer and collision gas. MassLynks MS (Waters, Milford, MA, USA) software v4.1 was used for method development and data acquisition, qualitative analysis and quantitative analysis. The multiple reaction monitoring (MRM) mode was selected to monitor the precursor-to-product ion transitions. The retention times and chromatographic parameters of pesticides are shown in Table 2.

### 2.6. GC–MS/MS Analysis

Pesticide residues were also analyzed by a gas chromatograph (Agilent GC 7890A, Agilent Technologies, Santa Clara, CA, USA) MS 7000 (Agilent) triple quadrupole equipped with two capillary columns (15 m length × 0.25 mm i.d. × 0.25 μm film thickness) and connected with a back flush system; helium was used as the gas carrier (1.4 mL min^−1^ column flow). Injection was set in a programmable temperature vaporizer (PTV) mode (initial injection 70 to final injection 280 °C), the source temperature was 230 °C. The column temperature was initially set at 65 °C for 1 min, raised at 30 °C min^−1^ to 100 °C × 0 min, 5 °C min^−1^ to 280 °C and maintained for 6 min. The injection volume was 3 μL in PTV solvent vent. Under the conditions described above, the retention time and further chromatographic parameters are reported in Table 2.

### 2.7. Statistical Analysis

Results were expressed as mean ± standard deviation of three replicates (*n* = 3) for each sample. Analysis of variance (ANOVA) was used to assess the effect of different processes on the pesticide, and Tukey’s test was used to establish the statistical significance (0.05) among samples. The data analysis was performed with XLSTAT software package v2016 (Addinsoft, New York, NY, USA).

## 3. Results and Discussion

### 3.1. Preliminary Evaluation of Pesticide Residues in Spinach

With the aim of evaluating, on an exploratory basis, the pesticide residues that can be frequently found in fresh spinach and frozen derived products, a preliminary evaluation was conducted on the S.A.L.P.A. S.A.C.arl’s database on the latest three years of crop production (2020–2022). Spinach samples, derived from standard (ST), integrated pest management (IPM) and organic (O) cultivation methods, were considered. The first cultivation method (ST) is not bound by any type of legislation except that of food safety defined by the European Union and corresponds to conventional agricultural production. The integrated pest management (IPM) has the purpose of ensuring the defense of productions by guaranteeing the lowest environmental impact in the framework of eco-compatible sustainable agriculture and refers to regional regulations. Finally, organic agriculture (O) is included in the cultivation methods governed by the reference legislation [30]. The preliminary results, mainly concerning ST products, highlighted that in the considered period (2020–2022), the propamocarb residue and its metabolite (propamocarb n-desmethyl) were identified on about 33% of the samples, with a mean concentration of ≈0.3 mg kg^−1^; while 23% and 24% of the samples contained lambda-cyhalothrin and chlorantraniliprole with mean values of 0.09 and 0.10 mg kg^−1^, respectively. In smaller percentages (5% and 6%) pyraclostrobin, fluopicolide and boscalid were also identified. Similar trends, in terms of detected substances and quantities, were found by analyzing the database as it referred to the two different crop seasons, i.e., summer (15/05–14/09) and winter (15/09–14/05). The results of this preliminary screening highlighted that all the identified residues were found to be up to 50 times lower than the maximum residual levels (MRLs) established for the spinach category and for each specific compound according to European regulations [31,32,33,34]. Based on the above discussed outcomes, four pesticides were selected and used for the experimental plan of this research work. Propamocarb, chlorantraniliprole, lambda-cyhalothrin and fluopicolide were selected; moreover, the presence of the propamocarb metabolite, propamocarb n-desmethyl, was also assessed in spinach samples.

### 3.2. Effects of Washing and Blanching Treatments for Pesticide Removal

**Figure 2:**
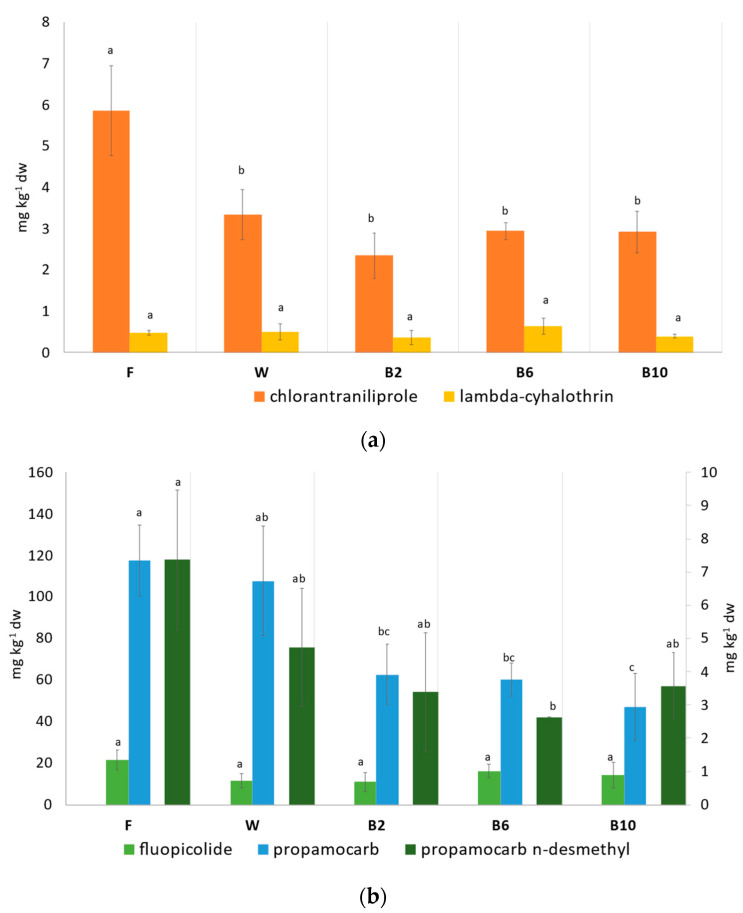
(**a**,**b**) Content of pesticide residues in treated spinach samples (mg kg^−1^ on dry weight). In (**b**), fluopicolide and propamocarb are reported on the primary axis, while propamocarb n-desmethyl is on the secondary axis. Different lowercase letters indicate significant differences between mean values of each residue concentration at different processing points (*p* < 0.05).

**Figure 3:**
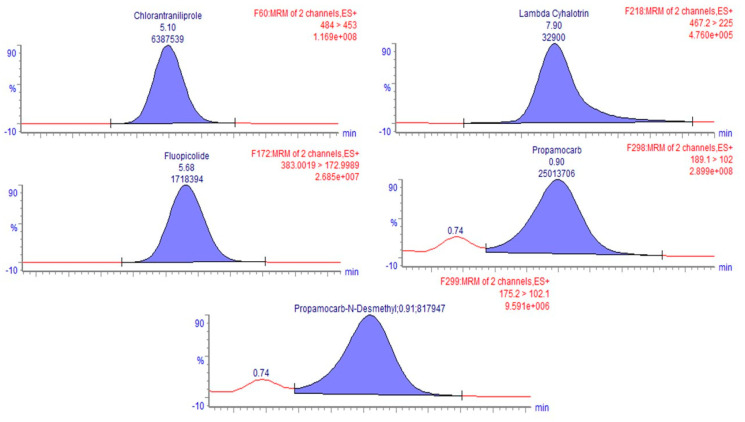
LC-MS/MS chromatograms of residues in fresh samples.

The concentration of the pesticides and the degradation products measured in spinach samples are shown in Figure 2a,b; chromatograms of residues of some replicates of fresh samples are reported in Figure 3, while those related to the treatments are shown in Figure S1. All the pesticide compounds were detected above the maximum residue levels (MRLs) in the raw samples (F), and therefore, it was useful to perform a study on PFs as also indicated by Bonnechère and co-authors [4]. The initial concentration of residues in fresh samples was 5.9, 0.5, 21.4, 117.5 and 7.4 mg kg^−1^ on the dry weight of chlorantraniliprole, lambda-cyhalothrin, fluopicolide, propamocarb and propamocarb n-desmethyl, respectively. Comparing the fresh samples with the treated ones, regardless of the type of operation used, a general reduction in the concentration of all the residues was observed for all the samples without any significant differences (*p* > 0.05) (Figure 2).

**Figure 4:**
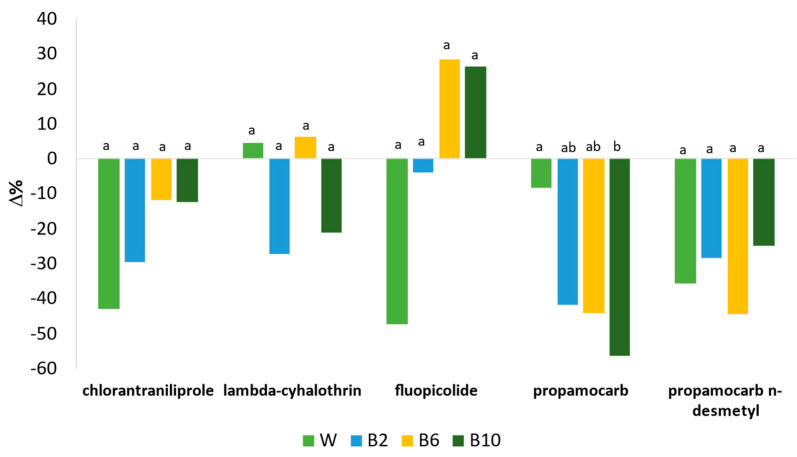
Variation (Δ%) of pesticide residues in spinach samples affected by different treatments. Different lowercase letters indicate significant differences between mean values of each residue for different processing (*p* < 0.05). Blanched samples are referred to the washed one.

Washing is considered the most common and straightforward form of processing. It is generally the first step in various types of treatments (household and commercial preparation) applied to food commodities [14]. In this study, the variation (Δ%) of pesticide residues at different operation steps is depicted in Figure 4. The washing step, operated with running water, led to a significant reduction of chlorantraniliprole (−43%) and fluopicolide (−47%), with respect to the fresh sample data; a slight reduction was observed for propamocarb (−8%) and propamocarb n-desmethyl (−8%), while conversely, a slight increase in lambda-cyhalothrin was depicted (+5%), although without significant difference (*p* > 0.05). As reported by Yang et al., washing with running water led to the highest removal efficiency among all methods used for different leafy vegetables; in spinach, this method accounted for about an 87% reduction in the pesticide content [35]. Different factors influence the persistence and the removal of pesticides; for example, polar, water-soluble pesticides, such as propamocarb, are more readily removed than low-polarity molecules [4,36]; furthermore, the systemic action mode, for which the active substance is absorbed into the system of a plant, renders its parts (the roots, stems and leaves) poisonous to plant pests and pathogens [4]. In this study, the small reduction, caused by the washing step, of propamocarb and the slight increase in lambda-cyhalothrin could be related to the systemicity and the hydrophobicity characteristics of the first and the second molecules, respectively. Furthermore, as reported by Wu et al., it was difficult to remove pesticides from spinach by tap water, showing a small reduction of lambda-cyhalothrin of 5%, when compared with other washing methods such as ozone water, detergents and alkaline solution [13]. The leaf characteristics (surface area, wax amount on cuticle, thickness) [35] and the age of chemicals could also be considered factors affecting the removal of pesticides; indeed, it was observed that pesticides were easily remove 1 day after spraying than 1 week after [37]. It is important to point out that the commercial pesticide formulations used in this study contained different coformulants that may have altered the kinetic degradation of each pesticide.

Concerning the blanching step, a decrease in residues was observed when 2 min treatment was applied (B2), compared to the washed samples. The highest reduction was depicted for propamocarb (−41%); about −30%, −29% and −27% reductions were observed for chlorantraniliprole, propamocarb n-desmethyl and lambda-cyhalothrin, respectively, while a small reduction was identified for fluopicolide (−4%). The reduction of propamocarb could be associated with the polarity of the molecules with the weakest log–octanol–water partition coefficient (−1.3 Log *p*), as observed also by Bonnechère et al. in blanched spinach [4].

Blanching for 6 min (B6) led to a decrease in propamocarb and chlorantraniliprole of −44% and −11%, respectively, while an increase in fluopicolide (+28%) and lambda-cyhalothrin (+6%) was observed. The extension of the treatment time to 10 min (B10) caused a decrease, with respect to the washed sample (W), of chlorantraniliprole (−13%), lambda-cyhalothrin (−21%) and propamocarb and its metabolite of −56% and −25%, respectively; conversely, an increase in fluopicolide (+26%) was observed. The controversial behavior of fluopicolide and lambda-cyhalothrin could be related to the log *p* value and the system of action of each compound, besides the operation conditions used, as the prolonged time of blanching (10 min) in an open pan. Concerning propamocarb content, the decrease is negatively correlated with the increase in the treatment time (−0.93). As also described and observed by Yang and co-authors, the prevalent physicochemical parameter affecting the rate of pesticide residue removal during heat treatment (blanching and boiling) was the partition coefficient [35]. In this study, the low partition coefficient (log *p*) of the propamocarb denotes its hydrophilic behavior, confirming the results reported by Nagayama that showed a negative correlation between the log *p* value and a reduction of residue content after blanching or making jam. Conversely, the higher the partition coefficient value is, the lower the pesticide reduction [38].

Concerning propamocarb n-desmethyl, a slight reduction was observed after 10 min of treatment with respect to 6 min, without any correlation. Despite the higher toxicity of the metabolite propamocarb n-desmethyl than the parent compound, to date, the toxicity values and physicochemical properties have not been studied, except in preharvest kinetic dissipation studies regarding cucumber, zucchini and tomato [39], both in water and soil [11]; therefore, it is quite difficult to infer the behavior of this compound.

Considering the residual concentration in the respective blanching waters, the highest content was observed for propamocarb and its metabolite, while lambda-cyhalothrin was not detected, regardless of the time of blanching applied, probably associated with the concentration effect in the spinach (B6) or because of the degradation or volatilization caused from the prolonged blanching (B10).

Furthermore, the different behaviors and fates of the pesticide compounds observed in this study could also be associated with different factors, linked to the process, such as the reduction of the intracellular water content, caused by the prolonged effect of blanching, resulting in a concentration effect of the compound [36] or due to the accumulation of pesticides as a result of moisture evaporation via heating in an open environment [40], as observed for chlorfenapyr in crow daisy for lufenuron in perilla leaves and ssamchoo (*Brassica lee* ssp. *namai*) after boiling (5 min 100 °C) [35], or for acetamiprid in green chilis after boiling [41].

Based on the obtained results, despite the fact that no significant differences were observed among the three blanching treatments, the optimal blanching condition seems to be 2 min treatment (B2) at 80 °C, which also reduces the negative effect of the blanching process over the vegetable structure.

The processing factor (PF), derived by the ratio of the residue concentration in the processed commodity to that in the RACs (raw agricultural commodities), was determined for all the residues with respect to the fresh sample to the washed one, and to the washed sample to the respectively treated ones (blanched). A factor < 1 (=reduction factor) indicates a reduction of the residue in the processed commodity, whereas a factor > 1 (=concentration factor) indicates a concentration effect of the processing procedures.

**Figure 5:**
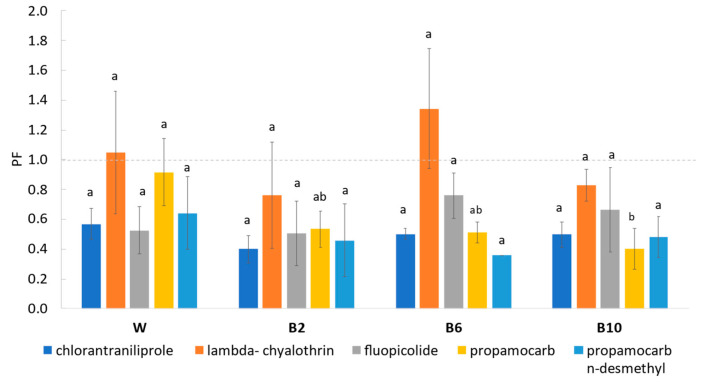
Pesticide residues’ processing factors (PFs). Different lowercase letters indicate significant differences between mean values of residue for each processing (*p* < 0.05). TF: <1: reduction of residue; >1: accumulation of residue.

Based on the results reported in Figure 5, chlorantraniliprole showed values lower than 1 at all the operation levels; lambda-cyhalothrin and fluopicolide showed variable accumulation behavior (W, B2, B6, B10) with values ranging from 0.7 to 1.3 and from 0.5 to 1.4 for the first and second compound, respectively, without significant differences (*p* > 0.05). Concerning propamocarb hydrochloride, PF values lower than 1 were observed, with significant differences only between the washed (0.92) and 10 min treated sample (B10) with a value of 0.4 (*p* < 0.05). Our results are consistent with previous findings from studies in spinach that showed for propamocarb a mean washing and blanching processing factor of 0.89 and 0.30, respectively, while a mean processing factor of 1.6 was shown for deltamethrin [4], similar to that detected for lambda-cyhalothrin in our study. Furthermore, the median values of processing factors reported in the EFSA database are similar to the processing factors for propamocarb (0.88) and lambda-cyhalothrin (1.7), despite that fact that no harmonized list of processing factors is available within Europe and worldwide [18].

Different PF values observed in this study could be associated with physicochemical properties of the compounds; indeed, as reported by Timme and Walz-Tylla, the persistence of pesticides in processed food depends on their octanol–water partition coefficient, which is an indicator of a pesticide’s hydrophilic or lipophilic properties and also to its higher tendency to accumulate inside the matrix [36]. As described by Bozena et al. for strawberry jam, the adsorption of the pesticides onto plant tissues and their solubility in water strongly influenced the removal of pesticide residues during the heat treatment process [42]. Therefore, as well-discussed in the literature, the characteristics of the pesticides, such as the solubility as well as the evaporation of water during the thermal process, may have influenced the residual amounts [35].

### 3.3. Effect of Frozen Storage on Pesticide Residue

The results of pesticide residue contents in frozen spinach samples, treated with different times of blanching (B2, B6, B10) and stored for 10 months at −20 °C, are presented in Table 3. Fresh and washed samples were not considered because the aim of the study was to assess the effect of the process and the content of residue in the final frozen product, ready for the consumer. Generally, no significant variations (*p* > 0.05) were depicted after the first day of frozen storage at −20 °C (t1) for all the pesticide residues, regardless of the time of blanching used, except for propamocarb n-desmethyl treated for 10 min (*p* < 0.05). It can be concluded that the freezing process, after blanching, did not influence the content of pesticides in spinach samples.

During the frozen storage period (0–300 days), important variations were depicted in all the treated samples. Chlorantraniliprole showed a significant increase (*p* < 0.05) in B2, B6 and B10 samples, reaching at the end of the storage (t300) values of 5.83 ± 1.11, 12.15 ± 2.00 and 11.94 ± 2.20 mg kg^−1^, respectively. Concerning lambda-cyhalothrin, an increasing trend was depicted, with a significant variation for B2, B6 and B10 samples during storage, starting mainly at t105 and ending at t300 with 4.17 ± 1.60, 6.38 ± 2.90 and 8.63 ± 0.30 mg kg^−1^,respectively, Fluopicolide content remained almost stable during frozen storage in B2 samples (*p* > 0.05), while a significant increase was observed at the end of the storage period for B6 and B10 samples. Propamocarb and propamocarb n-desmethyl showed similar trends. No significant variations (*p* > 0.05) were observed in B2 samples, and both the residue concentrations remained almost constant during the refrigeration period (0 to 300 days); B10 samples showed the same behavior observed in B2, while propamocarb n-desmethyl showed a significant reduction at t1, with a value of 1.23 ± 0.16 mg kg^−1^ compared to t0 and t300. Concerning B6 samples, a significant increase in propamocarb n-desmethyl was depicted in the sample stored for 300 days with respect to the initial content reaching a value of 3.87 ± 1.10 mg kg^−1^. Considering the influence of blanching time on the residue pesticide contents (Table 3), significant variations were highlighted at the end of storage period (t300) and mainly for chlorantraniliprole, lambda-cyhalothrin and fluopicolide, probably associated with the mechanical disruption of cells’ structure during processing, leading to a loss of cell vegetable water along with a dehydration effect of freezing [43] resulting in a concentration of pesticide molecules; conversely, this was trend not associated with propamocarb and its metabolite, for which the time of blanching did not significantly influence their content (*p* > 0.05).

Our results generally agree with the few presented in the literature, such as those observed in fish stored at −70 °C for four weeks for organochlorines, organophosphorus, pyrethroid and carbamates compounds [23] and for insecticides and fungicides in zucchini at −30 °C [24], where no significant reduction of pesticides was observed during frozen storage. On the contrary, reductions of difenoconazole residues were observed in mango at low storage temperature (−20 °C) [25]; in fresh, raw mackerel fillets at −20 °C for different pyrethroid pesticides [27]; in apples at −25 °C for fungicides [28]; and in tomatoes stored at −10 °C for organochlorines and organophosphorus compounds [26]. It is well-known that low temperatures reduce the chemical reactivity and decay of organic compounds. The storage at very low temperature of food contaminated with pesticides has been reported to have no significant effects on the pesticides’ levels [23]. Further, a sample’s matrix has abundant enzymes and microbes that can cause the degradation of the pesticide residue. Low storage temperature limits both the enzymatic and microbic activity that otherwise result in improved concentrations when higher storage temperature is used (−4 °C, +4 °C) [23,25]. The blanching treatments applied in this study could have inhibited the enzymatic activity, and therefore this degradation aspect cannot be considered. Furthermore, we should not exclude the effect of the freezing process on the texture modification of the spinach vegetable structure. Indeed, ice formation causes important damage in the plant tissue, influencing the dehydration, partitioning, concentration or degradation [44] that could affect the pesticide residue contents in frozen foods. Due to the limited number and controversial nature of existing studies, the main factors that affect the stability of the residues in frozen spinach remain unclear; even though the reactivity of pesticides by their molecular structure can be predicted, it is still difficult to know their degradation process, which depends upon the structure and transport behavior of pesticide, as well as the environmental conditions [9]. Furthermore, as previously discussed, the presence of coformulants may have modified the physicochemical interaction of the molecules with the vegetable matrix.

## 4. Conclusions

This study examined the effects of sequential processing operations such as washing, blanching, freezing and frozen storage on chlorantraniliprole, lambda-cyhalothrin, fluopicolide, propamocarb and propamocarb n-desmethyl content in spinach, considered as the main residues in spinach based on a preliminarily evaluation. The washing step was mainly effective for chlorantraniliprole and fluopicolide reduction and less on propamocarb and lambda-cyhalothrin content. Blanching was effective for the reduction of all the pesticides, regardless of the time used (2, 6 and 10 min). The freezing step did not influence the residue content, while during ten months of frozen storage, an increase in chlorantraniliprole, lambda-cyhalothrin, fluopicolide and propamocarb n-desmethyl was observed for the samples blanched for 6 and 10 min. No variation of pesticide contents was highlighted in frozen spinach samples blanched for 2 min. These findings highlighted the positive effect of washing and short-time blanching treatment (2 min) in pesticide residue content with positive effect also during spinach frozen storage. The knowledge of the physicochemical features, specific for each pesticide compound, represents an important point in order to optimize good agricultural practices (GAP) and the correct household or industrial food production treatments. Furthermore, research outcomes emphasize both the effects of frozen storage and the frozen products as possible health hazards due to pesticide content, opening this question to further studies because of the increasing consumption of frozen products, from a “field to fork” food safety perspective.

## Figures and Tables

**Table 1:** The main properties of pesticide contents in commercial formulations.

Commercial Product	Pesticide	Class and Mode of Action	MRLs mg/kg	Log *p* at pH 7, 20 °C	Water Solubility at 20 °C mg/L	Melting Point	Henry’s Constant at 25 °C Pa m^3^ mol^−1^	Stability *
VOLARE^®^	Fluopicolide 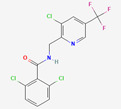	Benzamide and pyridine fungicides; systemic; absorbed by the roots and leaves and transported acropetally; modifies the distribution of fungal spectrinlike proteins.	6	2.9	2.80	150	4.15 × 10^−5^	Stable in tightly closed and dry conditions; slow degradation to photolysis and hydrolysis; degradation occurs through hydroxylation in the bridge carbon.
	Propamocarb hydrochloride 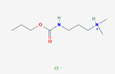	Carbamate fungicide; systemic; reduces mycelial growth and development of sporangia, influences the biochemical synthesis of membranes; absorbed by the roots and leaves and transported acropetally.	40	−1.3	1,005,000	64.2	8.50 × 10^−9^	Stable to hydrolysis, temperature up to 400 °C and photolysis.
ALTACOR^®^	Chlorantraniliprole 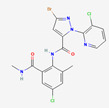	Systemic anthranilic diamide insecticide, it binds to ryanodine receptor, causing impaired muscle regulation, paralysis and insect death.	20	2.86	0.88	209	3.2 × 10^−9^	Fast aqueous photolysis; slow aqueous hydrolysis.
KARATE ZEON^®^	Lambda-cyhalothrin 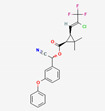	Pyrethroid insecticide with contact and stomach action and repellent properties; nonsystemic.	0.6	5.5	0.005	49.2	2.00 × 10^−2^	Stable to light, stable to storage >6 months at 15–25 °C and stable to decomp and cis–trans isomerization for at least 4 years in the dark at 50 °C.

* Information about stability was obtained from the 13th edition of the *Pesticide Manual: A World Compendium of Pesticides* [29].

**Table 2:** LC-MS and GC-MS parameters of identified pesticides.

Pesticide	Retention Time (min)	Precursor Ions (*m*/*z*)	Product Ions (*m*/*z*)	Cone Voltage	Collision Energy (V)	Quantifying Ions
**LC-MS**						
Lambda-cyhalothrin	7.95	467.2	225	16	23	x
		467.2	141.2	16	57	
Fluopicolide	5.68	383	173	40	20	x
		383	109	40	66	
Propamocarb hydrochloride	0.91	189.1	102	15	15	x
		189.1	144	15	10	
Propamocarb n-desmethyl	0.90	175.2	102.1	30	14	x
		175.2	144.2	30	8	
Chlorantraniliprole	5.12	484	453	20	14	x
		482	451	20	14	
**GC-MS**						
Lambda-cyhalothrin	33.55	181.1	152.1		30	
		181.1	127.1		35	

“x” identifies the quantifying ion.

**Table 3:** Content of pesticides residues (mg kg^−1^dw) in spinach samples treated with different times of blanching.

Blanching Methods	Storage (Days)	Residue Concentration (mg kg^−1^ dw)				
		Chlorantraniliprole	Lambda-Cyhalothrin	Fluopicolide	Propamocarb	Propamocarb n-Desmethyl
B2	t0	2.34 ± 0.55 ^a,B^	0.36 ± 0.17 ^b,D^	10.81 ± 4.68 ^a,C^	62.61 ± 10.45 ^a,A^	3.38 ± 1.79 ^a,AB^
	t1	2.57 ± 0.27 ^a,B^	0.31 ± 0.06 ^b,D^	12.61 ± 2.81 ^a,C^	77.93 ± 6.09 ^a,A^	3.83 ± 1.03 ^a,A^
	t105	2.93 ± 1.12 ^a,B^	2.93 ± 0.88 ^a,CD^	16.53 ± 10.44 ^a,C^	63.77 ± 21.80 ^a,A^	1.99 ± 0.11 ^a,A^
	t300	5.83 ± 1.11 ^a,B^	4.17 ± 1.60 ^a,BC^	24.85 ± 2.30 ^a,BC^	66.93 ± 10.30 ^a,A^	3.45 ± 0.40 ^a,AB^
B6	t0	2.94 ± 0.20 ^b,B^	0.64 ± 0.19 ^b,D^	16.23 ± 3.31 ^b,C^	60.09 ± 7.93 ^ab,A^	2.63 ± 0.10 ^b,AB^
	t1	2.30 ± 0.46 ^b,B^	0.24 ± 0.04 ^b,D^	11.40 ± 2.01 ^b,C^	43.86 ± 5.32 ^b,A^	1.62 ± 0.40 ^b,AB^
	t105	4.67 ± 1.51 ^b,B^	4.77 ± 1.37 ^ab,BD^	37.58 ± 1.04 ^ab,AB^	69.76 ± 10.66 ^ab,A^	1.67 ± 0.11 ^b,A^
	t300	12.15 ± 2.00 ^a,A^	6.38 ± 2.90 ^a,AB^	44.12 ± 1.00 ^a,A^	73.04 ± 11.90 ^a,A^	3.87 ± 1.10 ^a,A^
B10	t0	2.92 ± 0.50 ^b,B^	0.39 ± 0.05 ^c,D^	14.22 ± 6.04 ^b,C^	46.94 ± 16.09 ^a,A^	3.56 ± 1.10 ^a,AB^
	t1	2.37 ± 0.54 ^b,B^	0.33 ± 0.11 ^c,D^	8.53 ± 0.10 ^b,C^	36.04 ± 5.92 ^a,A^	1.23 ± 0.16 ^b,B^
	t105	3.88 ± 1.24 ^b,B^	4.11 ± 1.30 ^b,BC^	18.53 ± 8.28 ^b,C^	58.10 ± 14.59 ^a,A^	1.65 ± 0.45 ^ab,A^
	t300	11.94 ± 2.20 ^a,A^	8.63 ± 0.30 ^a,A^	45.58 ± 0.70 ^a,A^	78.61 ± 24.70 ^a,A^	4.05 ± 0.60 ^a,A^

Different lowercase letters in a column indicate significant differences among each blanching method during storage time (*p* < 0.05). Different uppercase letters in a column indicate significant differences among all the blanching methods during storage time (*p* < 0.05).

## Data Availability

Data available on request due to restrictions. The data presented in this study are available on request from the corresponding author.

## Supplementary Materials

The following supporting information can be downloaded at: https://www.mdpi.com/article/10.3390/foods12142806/s1, Figure S1: Example of LC-MS/MS chromatograms of chlorantraniliprole, fluopicolide, lambda-cyhalothrin (S1a) and propamocarb and propamocarb n-desmethyl (S1b) in spinach samples before (F) and after each treatment: washing (W), blanching 2 min (B2), blanching 6 min (B6) and blanching 10 min (B10).
