# Supplementary material for: The Effect of Washing, Blanching and Frozen Storage on Pesticide Residue in Spinach
Source: Foods. 2023 Jul 24;12(14):2806. doi: 10.3390/foods12142806 (PMC10379809; doi:10.3390/foods12142806)
Supplement: Supplementary file 1 [file foods-12-02806-s001.zip › foods-2507505-supplementary.pdf]

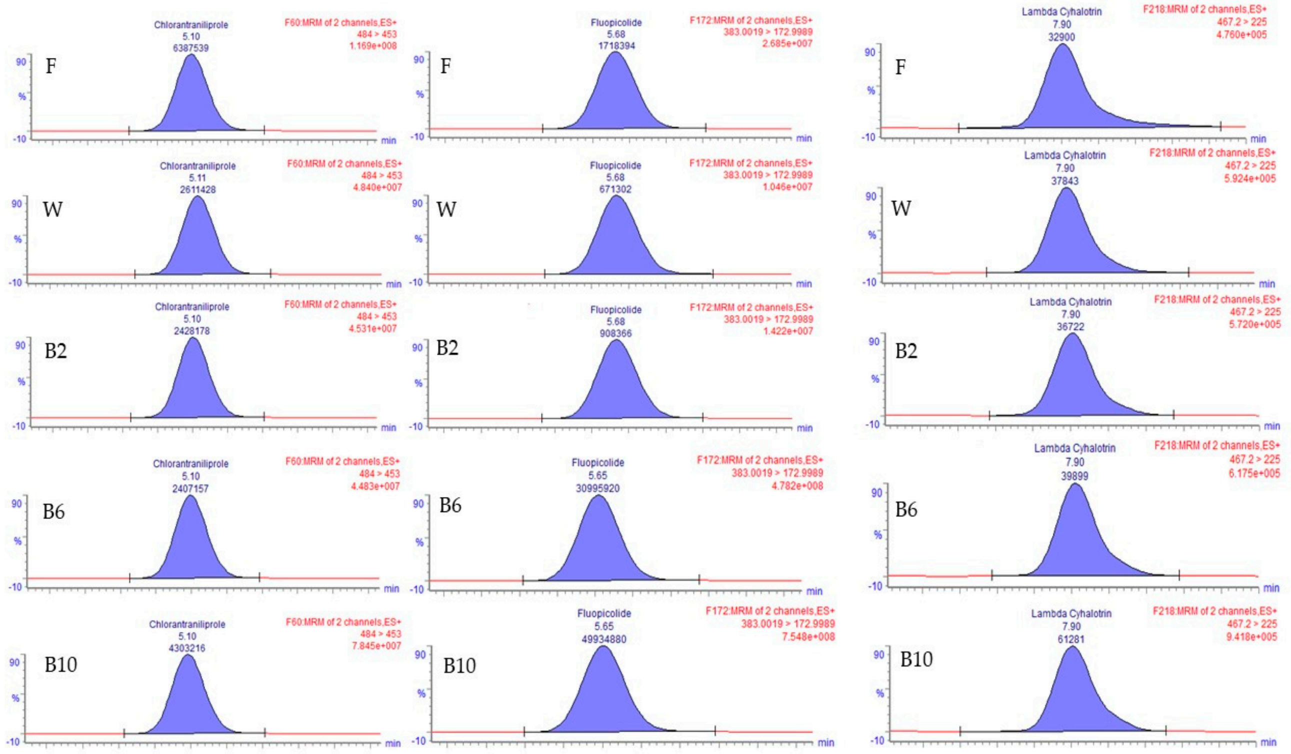

a

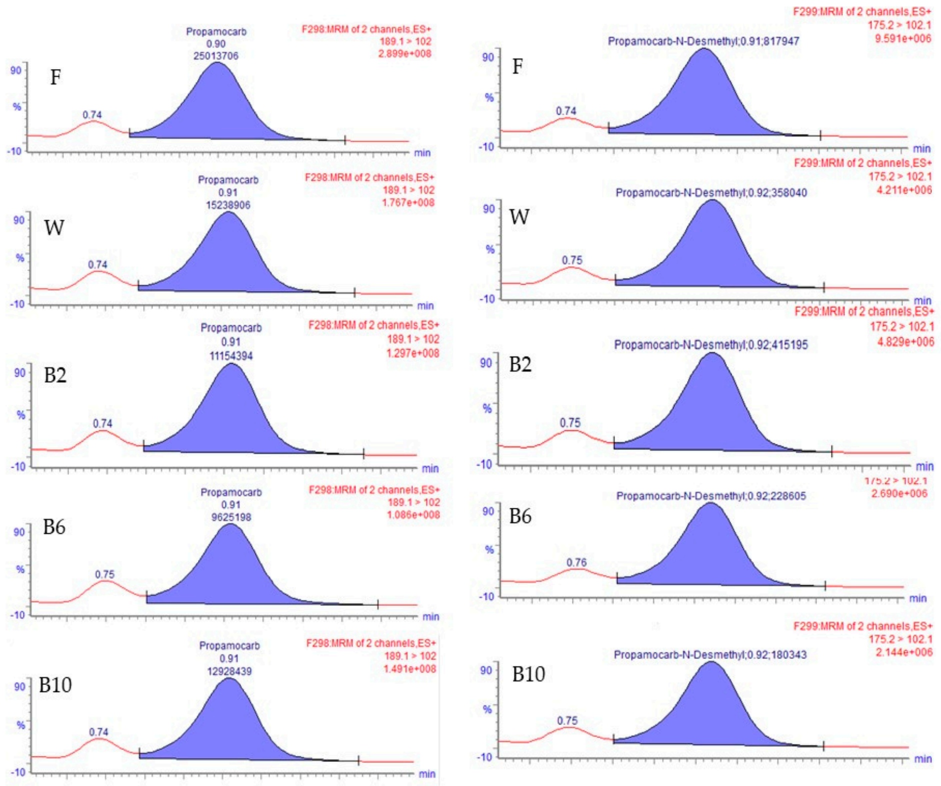

b

Figure S1 (a,b). Example of LC-MS/MS chromatograms of chlorantraniliprole, fluopicolide, lambda-cyhalothrin (S1a) and propamocarb and propamocarb n-desmethyl (S1b) in spinach samples before (F) and after each treatment: washing (W), blanching 2 min (B2), blanching 6 min (B6) and blanching 10 min (B10).
